# Recent progresses in novel *in vitro* models of primary neurons: A biomaterial perspective

**DOI:** 10.3389/fbioe.2022.953031

**Published:** 2022-08-17

**Authors:** Jiangang Zhang, Huiyu Yang, Jiaming Wu, Dingyue Zhang, Yu Wang, Jiliang Zhai

**Affiliations:** ^1^ Department of Liver Surgery, Peking Union Medical College Hospital, Chinese Academy of Medical Sciences and Peking Union Medical College, Beijing, China; ^2^ Departments of Neurosurgery, Peking Union Medical College Hospital, Chinese Academy of Medical Sciences and Peking Union Medical College, Beijing, China; ^3^ Departments of Orthopedics Surgery, Peking Union Medical College Hospital, Chinese Academy of Medical Sciences and Peking Union Medical College, Beijing, China

**Keywords:** *in vitro* model, primary neuron, biomaterial, scaffold, microfluidic chip, bioprinting

## Abstract

Central nervous system (CNS) diseases have been a growing threat to the health of humanity, emphasizing the urgent need of exploring the pathogenesis and therapeutic approaches of various CNS diseases. Primary neurons are directly obtained from animals or humans, which have wide applications including disease modeling, mechanism exploration and drug development. However, traditional two-dimensional (2D) monoculture cannot resemble the native microenvironment of CNS. With the increasing understanding of the complexity of the CNS and the remarkable development of novel biomaterials, *in vitro* models have experienced great innovation from 2D monoculture toward three-dimensional (3D) multicellular culture. The scope of this review includes the progress of various *in vitro* models of primary neurons in recent years to provide a holistic view of the modalities and applications of primary neuron models and how they have been connected with the revolution of biofabrication techniques. Special attention has been paid to the interaction between primary neurons and biomaterials. First, a brief introduction on the history of CNS modeling and primary neuron culture was conducted. Next, detailed progress in novel *in vitro* models were discussed ranging from 2D culture, *ex vivo* model, spheroid, scaffold-based model, 3D bioprinting model, and microfluidic chip. Modalities, applications, advantages, and limitations of the aforementioned models were described separately. Finally, we explored future prospects, providing new insights into how basic science research methodologies have advanced our understanding of the CNS, and highlighted some future directions of primary neuron culture in the next few decades.

## Introduction

Primary neuron culture is one of the most important techniques in the research field of the nervous system. Scientists first used traditional, Petri dish-based 2D cell culture approaches, which advanced the understanding of neuronal development in highly significant ways in the first few decades (Millet and Gillette, 2012). Ross Granville Harrison developed a revolutionary method to cultivate living tissues from vertebrates, which was the first time that primary neurons could be cultured *in vitro* (Gähwiler, 1999). Since then, the application of primary neuron has been expanded with the progress of our understanding of the nervous system.

Although the nervous system, especially the central nervous system (CNS) has long been a focal point of research, many questions remain unanswered. This could be ascribed to the complexity of the CNS in the human body. There are about 14–16 billion neurons and 61 billion supportive glial cells in a human brain, with different cells showing significant heterogeneity. These various types of cells co-regulate synapse formation, establish extensive neuronal networks, and the microenvironment around neurons (Herculano-Houzel, 2014), in which the extracellular matrix (ECM) could be remodeled constantly to form the macromolecular network, mechanically and biologically supporting the cells inside. Meanwhile, the ECM influences individual cell fates through physical structure, and as a result, causes changes in tissue architecture, circuit formation, and network function (Segel et al., 2019). Neurons, as the most important group of cells in the nervous system, possess a special structure. A neuron has an axon and several dendrites. Both axons and dendrites, collectively known as neurites, elongate the neuron, resulting in its characteristic shape (Petralia et al., 2015). While dendrites are responsible for building connections with surrounding cells by collecting signals from their highly branched protrusions, a neuron has only one axon to transmit its signal through its action potential. This communication between neurons and their downstream targets is typically achieved *via* synaptic electrical and chemical stimulation, ensuring the robustness of signaling pathways.

Studies on the nervous system require a faithful simulation of the *in vivo* microenvironment and cellular connection, which is the reason why the culture of primary neurons was proposed. The neurons are obtained from experimental animals, such as rodents, and then cultured in a Petri dish. However, such culture models could not meet the current need for research, as primary neurons cultured alone would show neither the interaction between neurons and other cells or the microenvironment around nor the real 3D tissue structure *in vivo*. With research going deep into the complex pathogenesis of various CNS and peripheral nervous system (PNS) diseases, and the new drugs targeting the CNS in process, it has been urgent to develop novel *in vitro* models of primary neurons.

A primary neuron culture model has a wide prospect of application. As mentioned earlier, it could be used to study the pathogenesis of CNS diseases like Parkinson’s disease and epilepsy (Lei et al., 2020; Milligan and Pachernegg, 2021), as well as peripheral nerve injuries. A more elaborate model, which is closer to the real *in vivo* condition, could be extremely valuable. For specific patients with CNS diseases, drug screening with such models could help clinical decision-making. Moreover, primary neuron culture made it possible to delve into the complicated mechanisms of the CNS and PNS, to advance our understanding of the structure and function of cells, tissues, and organs in the nervous system. There are also some applications intended for regenerative medicine, an emerging field of research, especially in spinal cord injuries, traumatic brain injuries, and peripheral nerve injuries. For such traumatic diseases, using neuro-regenerative therapies could promote neuronal recovery and improve patient outcome (Anjum et al., 2020).

Despite the pressing need and potential application of modified models of primary neuron, it would be difficult to design new models without new techniques. The development of biomaterials has been a critical factor. Traditional 2D cultures with Petri dishes were reductionist approaches, unable to explain the possible mechanisms and effectually predict clinical outcomes in humans. New biomaterials, such as hydrogels, have provided a versatile and tunable alternative (Walczak et al., 2021). It has a high water content and porous structure, which shows similar mechanical and biological properties as ECM. Hybrid hydrogels, comprised of blended polymers, enable even greater tailoring to modulate rheological factors, stiffness, shape fidelity, and other biomechanical characteristics. Biofabrication techniques make it possible to geometrically control the assembly of complex 3D structures from bottom to top, with the innovation of bioinks, scaffolds, and other functional elements (Song et al., 2020).

**FIGURE 1:**
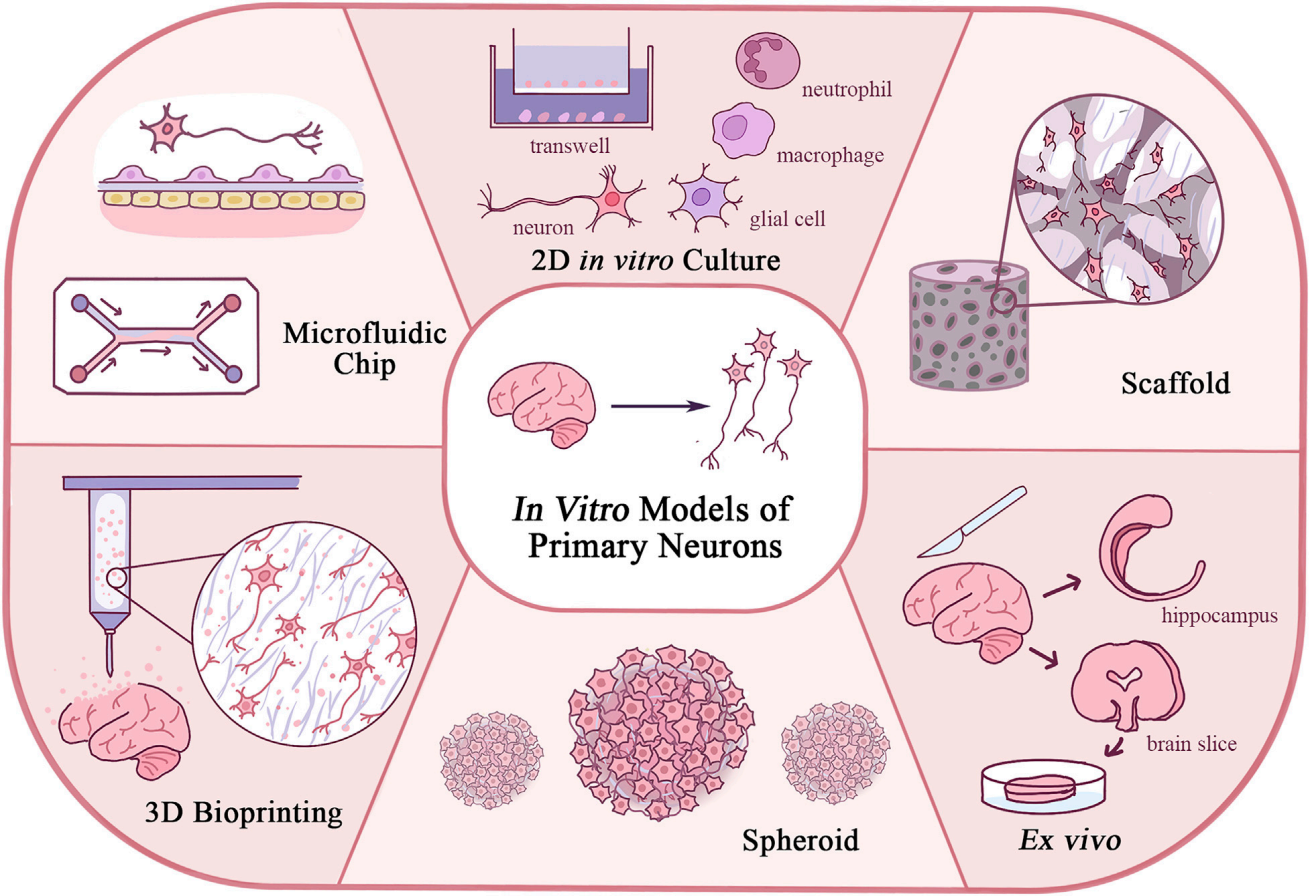
Schematic diagram of *in vitro* models of primary neurons illustrating features of each model.

Although the mechanical properties of biomaterials in neuron culture have not been fully understood, they are undoubtedly an important issue, and novel models could be developed based on the principle of regulating such properties (Walczak et al., 2021). This review explores the novel *in vitro* models with primary neurons developed in recent years, from the 2D *in vitro* model, *ex vivo* culture, and spheroid to 3D cultures as a scaffold, 3D bioprinting, and on-chip culture, especially focusing on the design and application of those models, and the new biomaterials involved (Figure 1). By introducing those models, we can have a holistic view of the development of primary neuron culture and how it has been connected with the revolution of biomaterials and techniques. Our discussion on the advantages and limitations of those different methods, on the other hand, might provide new insights into how basic science research methodologies have advanced our understanding of the CNS and PNS, and show some future direction of neuron culture research in the next few decades.

## 2D *in vitro* model

### Modalities

In the field of neurobiology research, 2D monolayer culture of primary neurons in a Petri dish remains the most commonly used *in vitro* model. With the perfected separation process of primary neurons and modified culture environment *in vitro*, primary neurons with high purity can be isolated from experimental animals and maintained under stable condition for a long time in a Petri dish coated with poly-lysine. Many of the molecular mechanisms have been confirmed by 2D *in vitro* models. However, with an increasing understanding of the nervous system, various neural cells were found to work collaboratively and thus, traditional 2D monocultures have undergone a transition to a more physiologically relevant multicellular and multi-chamber model. Compared with traditional monoculture, these novel *in vitro* models can better explore the direct and indirect interactions between different cell types including neurons, astrocytes, microglia, and other immune cells. In combination with gene manipulation techniques and inhibitors or activators for specific signal transduction pathways, a novel 2D *in vitro* model provides a convenient *in vitro* validation tool for intracellular signal transduction and intercellular communication under physiological and pathological conditions. Primary neural cells isolated from different animal models also meet the purposes of different studies. In addition, the survival of primary neurons and maintenance of normal physiological functions require support from adjacent cells through direct contact and paracrine. Therefore, co-culture of glial feeder cells and primary neurons could extend the survival of primary neurons cultured at low density and reduce oxygen tension (Kaech and Banker, 2006).

There were various methodologies to construct novel 2D *in vitro* models of primary neuron. First, neurons can be directly mixed with different types of cells and grown in Petri dishes in a predefined proportion to establish a 2D co-culture system of primary neuron. Since it takes a certain amount of time for neurons to adhere and develop neurites, neurons could be seeded in the Petri dish before other cells while other types of cells could be seeded at a desired time point (Natunen et al., 2019; Wang Y. S. et al., 2021). Similarly, the sandwich culture is another method to establish a 2D co-culture system of primary neurons and also creates a microenvironment of neuro-glial interactions. In a sandwich culture system, neurons and glial cells are usually cultured on the bottom of a Petri dish and cover glass, which can be placed into the dish upside down and separated from the bottom by an upholder such as a paraffin ball. In this way, neurons and other types of cells can be remained close and easily separated by removing the cover glass. According to different research purposes, the modified sandwich culture system has made it possible to observe fine subcellular structures and pathophysiological processes of primary neurons such as axon initial segments assembly and peroxisome deficiency (Abe et al., 2020; Yang and Bennett, 2021). Furthermore, Transwell assays have also been widely used in the study of interactions among different types of cells. The Transwell culture system consists of two chambers separated by a layer of porous polymeric membrane, in which different types of cells interact through paracrine. In the Transwell culture system, different types of cells are usually not in direct contact and could be easily separated, giving the model system greater flexibility for downstream neurobiology studies. Finally, it is convenient to utilize conditioned medium to study the effects of one type of cell on another. As one of the most simplified *in vitro* models, conditioned medium assay provides convenient tools for the insight of cell interactions such as the effects of astrocytes on neural progenitor cells (Faijerson et al., 2006). Novel 2D *in vitro* models simulate the interactions between neurons and other types of cells *in vivo* through different modalities. Although the development of biofabrication technologies enabled scientists to build a model with greater similarity to the microenvironment *in vivo*, a 2D *in vitro* model is still widely used in neurobiology research because of its simplicity and robustness.

### 2D Co-culture system of primary neuron

A 2D co-culture system of primary neurons established by directly mixing different types of cells or sandwich culture is suitable for exploring the interaction between neurons and glial cells, immune cells, and other different types of cells (Mai et al., 2020). For example, a 2D co-culture system of primary neuron and glial cells were applied in the study of neuro-glial synaptic connection (Wang Y. S. et al., 2021). A major application of 2D co-culture system of primary neurons is to study the effects of other types of cells on primary neurons under specific pathological conditions and to develop potential therapies. As models simulating ischemic stroke such as glutamate excitotoxicity, oxygen-glucose deprivation/reoxygenation (OGD/R), and peroxide insults could be easily built *in vitro*, and it has become commonly used to study the effects of astrocytes and microglia on neurons during ischemic stroke using 2D co-culture systems (Wen et al., 2021). The co-culture system was subjected to OGD/R using an anaerobic chamber and deoxygenated medium. Sandwich culture under OGD/R condition was helpful to study the effect of glial cells on neurons in ischemic-like circumstances. For example, sandwich culture or primary neuron and astrocyte under OGD/R condition revealed that NLPR6 may mediate the upregulation of inflammatory factors in astrocytes, thereby exacerbating neuronal damage (Zhang et al., 2020). There are similar application scenarios for 2D co-culture systems of primary neurons established by directly mixing different types of cells. The hippocampal neurons derived from embryonic C57BL/6 mice and the primary microglia co-culture showed that microglia exhibit toxic effects on neurons under ischemia stroke conditions. In addition, ganglioside GD3 knockdown microglia exhibited less neurotoxicity and impaired phagocytic function (Wang J. et al., 2021). In this study, primary microglia isolated from mice that had undergone global cerebral surgery and genetically manipulated mice were used to establish a 2D co-culture system, demonstrating the flexibility of cell types selected in these models. It is possible to simulate various circumstances *in vitro*, with the aid of a novel 2D co-culture system. Studies on the effects of microglia on primary neurons are not limited to ischemic-like conditions. Interactions between microglia and neuron have also been investigated in intracerebral hemorrhage (ICH) *in vitro* models. High dose of glutamate was used to simulate ICH and to generate neurodegeneration. Co-culture model of primary cortical neurons and BV12 microglia has revealed that activation of microglia leads to neural injury, and the potential neuroprotective and anti-inflammatory effects of small molecular compounds were evaluated (Yu et al., 2020). These applications indicated that the neuron-glia co-culture model is of great significance for drug development and pharmacokinetic research. Moreover, co-culture of neurons derived from different functional and structural areas of the brain also recreates the connections between different brain regions, which are important for pathophysiological processes such as learning and memory circuits and neurodegenerative diseases. For example, hippocampal and septal neurons derived from embryonic rat brains have been used to reproduce central cholinergic synapses *in vitro* and to further analyze their subcellular structure (Djemil et al., 2021).

Furthermore, primary neuron co-culture models are important for assessing the neurotoxicity of biomaterials such as nanoparticles. The neurotoxicity of biomaterials requires full evaluation as their applications in brain imaging and drug delivery to the CNS are in rapid development. Primary cortical neurons and microglia co-cultures from newborn rats were used to assess the neurotoxicity of nanoparticles (Shin et al., 2021). The co-culture model including microglia treated with silica-coated magnetic nanoparticles witnessed enhanced neurotoxicity and D-serine secretion, resulting in the accumulation of inclusion bodies in primary cortical neurons. It was suggested that some of the nanoparticles could mediate toxic effects on neurons through microglia. In addition to nanoparticles, the neurotoxicity of commonly used chemotherapeutic drugs such as platinum-based drugs have also been evaluated in primary neuron co-culture systems, in which astrocytes have demonstrated a neuroprotective effect mediated by mitochondrial transfer (English et al., 2020). In conclusion, 2D co-culture system of primary neurons plays an important role in the modeling of diseases such as ischemic stroke and intracerebral hemorrhage and can be used to study neurotoxicity and other interactions between neurons and glial cells.

### Transwell culture

Transwell cultures, which still rely on 2D culture of neural cells, simulate microenvironments of the nervous system *in vitro* efficiently. By adjusting the types and distribution of cells loaded within the two chambers and optimizing biomaterials, including changing the pore size of the polymeric membrane and coating biomaterials, functional structures in the nervous system with great complexity could be restored, for example, the blood–brain barrier (Stone et al., 2019). The two chambers can simulate environments on different sides of BBB. Endothelial cells, astrocytes, pericytes, and neurons seeded on the apical side of the membrane, the underside of the membrane and the plate bottom simulate the structure of BBB *in vitro*. The function of BBB can be restored by optimizing the type, proportion, and distribution of cells. In addition, Transwell cultures offer valuable cues for the interaction between primary neurons and glial cells in mechanical injury, ischemic stroke, and other pathological conditions (Liang et al., 2021; Lorenzen et al., 2021). The porous polymeric membrane structure was also utilized to study novel cell–cell interaction modalities, such as extracellular vesicles and small RNA-based intercellular crosstalk (Mao M. et al., 2020). For example, Transwell cultures revealed that primary hippocampal neurons transfer pathological α-synuclein to astrocytes through exosomes under METH exposure to mediate inflammatory response (Meng et al., 2020). Due to the existence of a porous membrane, cells cultured in two chambers interact through substances that can pass through the insert such as exosomes. This feature of Transwell culture allows precise control of variables and provides convenience for the study of indirect interaction between different cells. Similar to 2D co-culture systems, Transwell cultures are also capable of exploring the effects of potential drugs on primary neurons in conditions that simulate *in vivo* microenvironments. For example, anti-inflammatory effects related to the improvement of Parkinson’s disease symptoms were shown, and the underlying mechanisms were further explored in Transwell cultures, which serve as a part of preclinical studies and an effective supplement to animal experiments (Lei et al., 2020). Primary neurons were injured by 6-hydroxydopamine to simulate Parkinson’s disease, and indirect interaction between astrocyte and neuron was investigated. In addition, with the aid of Transwell culture of astrocyte and primary neurons, astrocytes overexpressing TDP-43 showed an important role in neurodegenerative diseases, and the model further contributed to the study of molecular mechanisms and the development of potential therapeutic drugs (Lee et al., 2020). In short, Transwell cultures are widely used as supplements to the 2D co-culture system due to the convenience to study indirect cellular interactions, the simplicity of cell separation between upper and lower chambers, and the precise control of variables.

### Advantages and limitations

As mentioned earlier, the 2D co-culture system and Transwell culture are still the most widely used multicellular *in vitro* models, with primary neurons due to their convenience, robustness, and flexibility for subsequent analysis. With the maturation of molecular manipulation technologies and system optimization, many finely regulated cell interaction mechanisms have been discovered (Gao et al., 2019; Ioannou et al., 2019). Novel 2D co-culture models with greater complexity have also been established, such as primary neurons, astrocytes, and microglia tri-culture models, to better simulate the *in vivo* microenvironment (Goshi et al., 2020). However, there are limitations regarding to the 2D co-culture system and Transwell culture. As for the 2D co-culture system, it is difficult to isolate different types of cells, which complicates subsequent characterization experiments. Transwell culture, on the contrary, is not suitable for all study purposes due to the absence of direct contact between different types of cells despite its flexibility and convenience. Moreover, the 2D co-culture system and Transwell culture still belong to 2D culture systems, which lack the mechanical support and biochemical cues provided by 3D structures and ECM in 3D culture systems. Cell morphology and phenotype of these models are significantly different from cells *in vivo* because cells cultured in these models are still attached to a 2D surface. Therefore, the 2D co-culture system and Transwell culture are inferior to 3D culture systems in terms of biological relevance.

On the other hand, the improvement of biomaterials exerts a great impact on the 2D co-culture system and Transwell culture. Primary neural cells are sensitive to the extracellular environment such as matrix stiffness, bio-reactive substances, and microstructure of biomaterials. Minor changes in biomaterials applied in the 2D co-culture system and Transwell culture will lead to a huge impact on primary neurons and thus, the development of novel biomaterials also exhibits great potential in the treatment of neurological diseases and *in vitro* modeling of those diseases. For example, a different culture system can be obtained by replacing poly-lysine, which is usually used to coat Petri dishes in 2D monoculture, with Matrigel (Karahuseyinoglu et al., 2021). Compared to the traditional monoculture, a culture system using Matrigel witnesses the formation of many clusters of neural cells with extensive neurites connections. Cell morphology, immunofluorescence staining result, and patch clamp-based electrophysiological recording showed significantly distinct network structures when biomaterials were replaced. The usage of novel biomaterials such as Matrigel can be regarded as a bridge between the traditional 2D models and 3D culture system since cells are embedded in biomaterials and extended in three dimensions. The changes in the growth pattern and electrophysiological capacity induced by novel biomaterials require further characterization to comprehensively validate specific *in vitro* models.

## *Ex vivo* model

### Modalities

With the development of 2D culture of primary neuron, complicated mechanisms could be studied using the co-culture system and special flasks. This still, however, did not solve the limitation that these systems were not able to simulate the distribution of cells *in vivo*, especially in 3D structure. Therefore, *ex vivo* models were adopted. The term “*ex vivo*” refers to a situation where tissue or part of an organ is removed from the body and preserved in culture. In this model, tissue sections were cut by a tissue chopper under precise parameters including the cutting range and thickness of slices. They were then being cultured on thin film inserts at gas–liquid interface (Sekizar and Williams, 2019). The slices could be 200–500 μm in thickness, and currently, the most commonly used biomaterial for slice cultures is polytetrafluoroethylene (PTFE), for the preparation of porous membrane. Other vessels using special biomaterials were also designed for specific experimental use, such as indium tin oxide glass coatings for thermal stability and the glass-bottomed Petri dish for fluorescence microscopy (Millet and Gillette, 2012).

### Brain slice culture

Brain slice *ex vivo* culture could be used for studying the mechanism of the connection between neurons and surrounding cells, neurons and their microenvironment such as myelination and synaptic responses. Although novel models are making much better duplication of the human CNS, its complexity has still made it impossible to demonstrate all synaptic responses and nerve fiber connections. Therefore, *ex vivo* culture makes up for the deficiency of 2D *in vitro* cultures in the past. Hippocampal slices were used to study the possible mechanism of leucine-rich glioma-inactivated 1 (LGI1), a secreted transsynaptic protein. Injection of shRNA-LGI1 in the hippocampus increased the excitability of dentate granulosa cells and the low-frequency facilitation of mossy fiber transmission to CA3 pyramidal cells. These results indicated that the *ex vivo* model could be applied to study the mechanism of neural interaction, with synaptic plasticity to some extent, and the model is easy to modify, which might lead to the finding of new pathways in treatment of diseases such as limbic encephalitis and temporal lobe epilepsy (Lugarà et al., 2020). In addition, *ex vivo* models could be used to understand the neuron crosstalk. In an *ex vivo* primary culture model of CNS of *medicinal leeches*, researchers found that microglia produced a large number of exosomes and presented strong interactions with neurites in primary neuron culture (Raffo-Romero et al., 2018). This finding promoted the research of exosomes in the crosstalk between microglia and neurons. With slices of brain, cerebellum, and spinal cord for *ex vivo* primary culture in a Petri dish, researchers could establish a 3D culture system for myelination, demyelination, and remyelination to replace the co-culture system of oligodendrocytes and neurons and to simulate the 3D cell structure *in vivo* (Sekizar and Williams, 2019). This is more economical and less difficult for live imaging, compared to *in vivo* studies.

Pharmacological research is another vast field of application. In organotypic *ex vivo* hippocampal slice culture, N-acetyl-serotonin (NAS) prevented cell death induced by oxygen-glucose deprivation or H_2_O_2_, and it also reduces hypoxia/ischemia injury in the middle cerebral artery occlusion mouse model (Zhou et al., 2014). Research on levetiracetam (LEV) explored whether it enhances the impact of a rate-limiting mechanism in vesicle trafficking termed supply rate depression on excitatory presynaptic terminals. By measuring monosynaptic connections with electrophysiologic assays in *ex vivo* hippocampal slices from wild-type and synapsin knockout mice and other *in vitro* models, scientists confirmed the enhancement of LEV at Schaffer collateral synapses (García-Pérez et al., 2015).

Most brain slices were cut from the hippocampus because of its cell function and convenience of extraction; meanwhile, slices of other brain tissue were also explored. The six-layered structure of the cerebral cortex is generated in an inside-first, outside-last manner, in which the first-born neuron stays close to the ventricle, while the last-born neuron moves past the first-born neuron to the surface of the brain. Therefore, a key process in normal cortical function is the regulation of neuronal morphogenesis. In an *ex vivo* research, techniques for analyzing neuronal migration and morphogenesis of organotype slices of the cerebral cortex were introduced to understand how neuronal morphogenesis is regulated in the tissue environment using RNAi electroporation (Lizarraga et al., 2012). Since only a small group of cells expressed the RNAi constructs, the organotypic slices were allowed for a mosaic analysis of the potential phenotypes, which provided a low cost and rapid alternative for transgenic or knockout animals with unknown cortical function.

### Hippocampus culture

Some specific parts of the brain could be cultured *ex vivo* for more structural research besides slice cultures. Such model was particularly widely used in research focusing on the development of the CNS. The most developed model was still applied in hippocampal research. For fetal alcohol spectrum disorder (FASD), a major non-genetic cause of developmental delay, which has currently no treatment, an *ex vivo* model was involved to compare the expression of insulin signaling pathway genes in fetal hippocampus *in vivo* and primary hippocampus culture *ex vivo*, based on the known role of the insulin signaling pathway in hippocampal function. Males and females showed different hippocampal transcriptional profiles during prenatal treatment, and there were also gender differences in Igf2 and Insr expressions (Tunc-Ozcan et al., 2016). This suggested that the effect of fetal ethanol concentration on gene expression of hippocampal insulin pathway is parallel *in vivo* and *ex vivo*, and the similarity of the changes in gene expression of prenatal ethanol dehydrogenase reactivity under those two conditions confirms that these effects are already set in the fetal hippocampus at GD18. The research strengthened the feasibility of the primary hippocampal culture model, which could be applied in drug detection and screening of FASD. Such a parallel experiment design could help understand the imperfection of *in vitro* models by comparing the result of *ex vivo* and *in vitro* cultures.

### Advantages and limitations

The brain slices and tissue compartments could be easily made, and the cellular structure of the tissue could be easily preserved, offering the possibility of performing several types of studies with simplified methodology and low cost. It also allows for precise adjustment of the cell culture environment. Studies based on such models are useful for exploring the correlation between structure and function, as well as the plasticity of neuronal interactions under normal and pathological conditions. Moreover, it may be used for genetic testing and molecular screening in basic and translational research (Lossi and Merighi, 2018). The convenience of this technique, on the other hand, has led to the limitation of its further development. Brain tissues could survive *ex vivo* culture, but they could not be preserved under good condition for a relatively long period despite the perfusion systems, membrane inserters, and culture chambers being developed and evolved to improve tissue activity. The heterogeneity of experimental animal models and the condition of drawing materials would also limit the reproducibility and general applicability, so standardized models could be hard to build up.

## Spheroid-based model

### Modalities

With the increasing understanding of the complexity of the CNS, *in vitro* models with primary neurons have gradually changed from the traditional 2D culture to the 3D culture system that is more relevant to the microenvironment *in vivo*. The development of biomaterials and biofabrication technologies has promoted the exploration of various 3D culture systems. Spheroid-based models are independent of scaffold biomaterial and have been broadly used in the fields of disease modeling and drug screening (Sirenko et al., 2019; Heinrich et al., 2021). The basic principle of constructing a spheroid-based model is to take advantage of the capability of cells to spontaneously form 3D aggregations. Biotechnologies utilized to establish a spheroid-based model have been developed such as hanging drop cultures, matrix embedding, high-density cell seeding, and low-attachment surface. The formation of cell aggregations mainly relies on the interactions among primary neural cells and ECM produced by neural cells themselves. As primary neurons are very sensitive to the microenvironment, nuances in biomaterials used in neurosphere models could lead to significant changes in cell functions as well as morphologies. Therefore, the impact of biomaterials on primary neurons in a spheroid-based model is also one of the most important research interests of *in vitro* modeling and regenerative medicine. Similar to the neurosphere model, organoid culture based on stem cell-derived neural cells and Matrigel enabled better reconstruction of the development process of the CNS. Brain organoids formed organ-like microstructures with spatial heterogeneity and functional features (Lancaster et al., 2013; Schukking et al., 2018; Pollen et al., 2019), which are one of the hotspots in neurobiological research. However, the discussion of organoids is beyond the scope of this review since neural organoid was usually derived from stem cells.

### Primary neurosphere model

Compared to traditional 2D models, the primary neurosphere model preserve cellular heterogeneity to a greater extent and exhibit many characteristics that are distinct from 2D cultured primary neuron. For example, primary neural cells derived adult *Prairie voles* aggregated to form neurospheres under low-adherent culture conditions and showed satisfactory viability (Avila-Gonzalez et al., 2020). Combining rat primary neurons and microglia cells with human brain microvascular endothelial cells, pericytes and astrocytes, and neurospheres with multiple cell types that mimic the blood–brain barrier (BBB) were constructed *in vitro*, using agarose gel culture and ultralow attachment plates (Kumarasamy and Sosnik, 2021). The heterocellular neurosphere formed a neurovascular unit and restored some of the key structures and functions of the BBB. This complex multicellular neurosphere model was suitable for studying the permeability of new drug delivery biomaterials such as nanoparticles to BBB, which is conducive to the development of nanomaterials for clinical application. Neurospheres were also successfully established in a collagen-based 3D culture system using primary cortical neurons derived from NRG1-knockout mice, which were reactive to electrical stimulations (Zhang et al., 2018). In addition, the cell morphologies, as well as phenotypes of primary neurons, were significantly affected by different biomaterials, such as silk scaffold, collagen, and Matrigel (Tang-Schomer et al., 2018), which is discussed in the section of the scaffold-based model. Other novel biomaterials such as cellulose exopolysaccharide derived from sugarcane molasses, collagen hydrogel with the addition of small molecules, and thermo-reversible gelation polymer have also been used for the establishment of 3D primary neurosphere model (Yang et al., 2009; Cruz Gaitán et al., 2015; Goncalves-Pimentel et al., 2018), which is helpful to explore the potential application of novel biomaterials regarding regenerative medicine. Moreover, the mammalian olfactory receptor neurosphere model has also been successfully established and achieved long-term maintenance under specific culture conditions based on recombinant proteins (Kim S. et al., 2018). The popularization of such a primary neurosphere model will be helpful to the study of specific neurons subtypes and their application in bioengineering. Furthermore, novel bioengineering techniques such as air–liquid interface (ALI) culture are expected to enhance the stemness of primary neural cells (Usui et al., 2017) and achieve fine regulation of the differentiation process.

### Advantages and limitations

Primary neurosphere models possess 3D spatial structures while neurites extend in all directions instead of being confined to the 2D plane of a Petri dish, which is significantly different from the traditional 2D models. Furthermore, primary neurosphere models make it possible to conduct *in vitro* experiments that rely on 3D spatial structures such as the study of micromotion around neural implants (Spencer et al., 2017), which served as an important supplement to animal experiments. Compared to other 3D culture systems, primary neurosphere models are simple to establish and have good potential for high throughput application, which is vital for preclinical drug screening. Meanwhile, characterization methods for primary neurosphere models such as optical clearing methods used in 3D imaging and oxygen probe in neurospheres have been gradually optimized to popularize the applications of primary neurosphere models (Dmitriev et al., 2014; Boutin and Hoffman-Kim, 2015). Regarding the source of neurons, primary human neurons are extremely difficult to obtain at present, and the sources are limited to surgically resected brain tissues. Therefore, there are few neurosphere models established from primary human neural cells. Stem cell-derived neurons rather than primary neurons are more commonly used in current spheroid- and organoid-based models (Son et al., 2008). Stem cell-derived organoid can better simulate the embryonic development process of the CNS and pathogenesis of CNS diseases such as Parkinson’s disease (Chumarina et al., 2019). Primary neurosphere models mainly rely on the interaction and adhesion among cells; therefore, it is difficult to control cellular arrangement within the spheroid. Neural cells in the primary neurosphere model lack organ-like cell arrangement and fine structures of the neural cells *in vivo*, remaining inconsistency is with the internal environment. Moreover, the types of cells involved in the formation of neurospheres are limited, and the homogeneity among different spheroids cannot be guaranteed. In order to realize the construction of more biologically relevant *in vitro* models, the aid of other bioengineering technologies is urgently needed.

## Scaffold-based model

### Modalities

**FIGURE 2:**
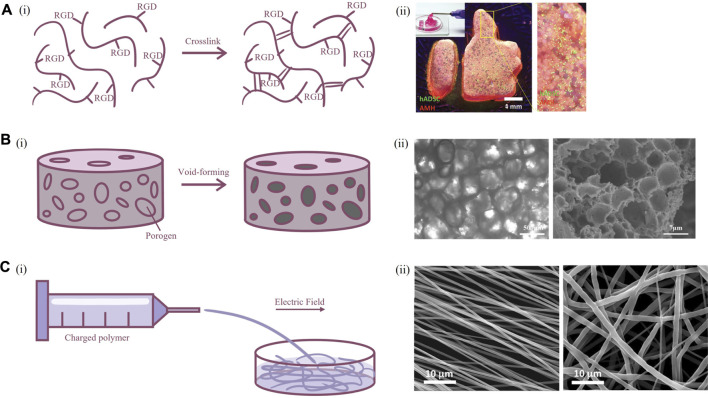
Fabrication techniques of scaffold for primary neuron culture. **(A)** Hydrogel scaffolds. **[(A), i]** Schematic illustration of crosslinking of modified polymers. **[(A), ii]** Image of hydrogel scaffold (Hsu et al., 2019). **(B)** Porous scaffolds. **[(B), i]** Schematic illustration of porogen emulsion and phase separation. **[(B), ii]** Bright-field microscope and scanning electron microscopy image of porous scaffold (Zhu et al., 2020). **(C)** Fibrous scaffolds. **[(C), i]** Schematic illustration of electrospinning. **[(C), ii]** Scanning electron microscopy of scaffold formed by aligned and random electrospun fibers (Amores de Sousa et al., 2020).

Scaffolds are the most fundamental 3D culture system. The narrow definition of scaffold-based culture included in this paragraph refers to the fabrication of a biological frame followed by the filling of cells. Compared with traditional 2D culture systems, scaffolds can provide cells with appropriate 3D environmental characteristics, attach points to maintain their *in vivo* morphology, and they are tractable in spatial arrangement to form a tissue-like structure. Biomaterials for scaffolds can be divided into natural polymers, such as gelatin, alginic acid, silk, chitosan, and synthetic polymers, such as poly-ε-caprolactone (PCL), polylactic acid (PLA), polyglycolic acid, and the combination of both (Murphy et al., 2017). The structures of scaffolds are mostly hydrogels or solid fibrous/porous scaffolds (Figure 2). One of the necessary characteristics of biomaterials for cell culture is biocompatibility. As synthetic polymers tend to lack cell-matrix binding sites, they often require modification of certain chemical groups to ensure the viability of the cultured cell. Although natural polymers usually have advantages on biocompatibility, it may still be adjusted on account of specific preference for interaction sites in different cell types (Kim et al., 2020). Scaffold construction is a multidisciplinary field including photochemical crosslinking, molding, 3D printing, chemical vapor deposition (CVD), and electrospinning. Optimal methodology is selected based on material properties and application requirements. Such plasticity can adapt to the disposition of different types of cells; therefore, scaffolds have a very widespread application and significant research value.

### Scaffold-based culture of primary neurons

Cultivation of nerve cells is particularly demanding. Neurons are terminally differentiated cells that cannot undergo regeneration. Living neurons continuously initiate and transduce electrical signals in the body and form synaptic connection with each other to constitute the complex neural network of the human brain. This requires massive energy and makes neurons highly sensitive to the lack of nutrients. In the meantime, the effective functioning of neural networks requires the abundant assistance of stromal cells and ECM; hence, the crucial significance of scaffold is to simulate these supports *in vitro*.

The mechanical properties of scaffolds are very important for neuron culture. Brain tissue is relatively soft, and this stiffness-related mechanosensory is vital for elongation of axon and normal development of the nervous system (Koser et al., 2016). Use of soft material to cultivate micropatterned neurons can promote rapid formation of a neural network structure (Lantoine et al., 2016). Neurons cultured on softer collagen had similar Na^+^ current as neurons *in vivo* than neurons cultured on glass (Evans et al., 2019). However, in order to restore the environment that neurons subsist in, it is necessary to ensure the stability of the scaffold and provide enough porosity to allow the infiltration of nutrients and communication between neurons. Silk proteins are biological macromolecules extracted from cocoons with suitable mechanical properties, great plasticity, and controllable biodegradability (Huang et al., 2018). Since the compressive modulus of silk protein scaffolds is higher than that of brain tissue, combining silk proteins and collagen could produce a scaffold with a relatively soft feature and enough shape fidelity that resemble the mechanical environment of brain tissue, providing long-term survival of neurons *in vitro* (Tang-Schomer et al., 2014). Scaffolds constructed using silk protein and collagen can promote primary neuron adhesion, axon elongation, and synaptic formation compared to 2D culture and can be further applied in the study of neuro-microglia interactions (Zhu et al., 2020). Similarly, the combination of natural and synthetic polymers, as the copolymer formed by chitosan and oligo-lactide, can be used in the culture of neurons to reduce the cytotoxicity of chitosan and produce mechanics more suitable for implantation (Grebenik et al., 2020). From another perspective, permeability of scaffold material could be enhanced by incorporation of microporous, which had additional benefits in promoting axonal growth (Hsu et al., 2019). The porous structure could be easily produced by mixing biomaterials with place-holding spheres or shapes with an appropriate size, which could be later removed to form a void within scaffold (Huebsch et al., 2015). The materials for place-holding should possess good shape fidelity during fabrication of scaffold, and resolved or melted under culture conditions, for example, poly (ethylene oxide) in the printing of gelatin methacryloyl (Ying et al., 2018).

Biocompatibility is another focused topic in the scaffold field. The cell-matrix interaction is carried out by the binding of receptors on the surface of the cell membrane and ligands in the ECM, which activates downstream signals that play a critical role in the regulation of cellular morphology and function. Strategies for improving biocompatibility include modification of the surface chemical group or microstructure. Synthetic and some natural polymers lack cellular binding sites, so they need to be modified with short peptide sequences, such as RGD, IKVAV, and YIGSR, which are common binding sites for neurons. RGD (Arg-Gly-Glu) is the short peptide ligand of integrin receptor and is the most well-known short peptide sequence in biomaterials science. Alginate scaffold modified with RGD could assist neuronal culture with higher function than unmodified scaffold (Kim et al., 2020). Laminin is one of the most important components of the basal membrane in the brain, while IKVAV and YIGSR belong to the α1 and β1 chains of laminin, respectively. Neurons cultured on PCL fibers functionalized with laminin showed better activity, higher number of neurons, and more uniform orientation of dendrites than neurons cultured on PCL fibers modified with RGD (Amores de Sousa et al., 2020). As some cytokines regulate neuronal growth, they can be immobilized with scaffold by biotin–streptomycin bonding, which can promote neuron growth even better than soluble cytokines (Kunze et al., 2011).

In addition, the surface microstructure of biomaterials also affects the phenotype of neurons. A neuro-templated scaffold was developed from silicification and calcination of the 2D cultured neural network, and neurons cultured on this biologically inert neuro-templated topography have significantly elongated axons (Kim B. J. et al., 2018). More simplified designs, such as polydimethylsiloxane (PDMS) scaffolds with dense micropillars on the surface, can also promote the maturation of neurons and be used for the construction of brain injury models (Chen C. et al., 2019). Likewise, the adhesion and survival of neurons could be enhanced in micropillar PCL scaffolds, facilitating the formation of a highly interconnected cell network. The scaffolds can also be used as a sustained release system of brain-derived neurotrophic factor, further upregulating the formation and function of synapses, and showing potential as implants (Limongi et al., 2018).

The electrical conductivity of the scaffold should also be taken into consideration. The most important phenotype of neuronal function is the generation and conduction of electrical signals; and this process requires the assistance of various cells and matrix in the physiological state. How to resemble the electrophysiological characteristics of neurons *in vitro* is the key to the authenticity of the *in vitro* model. Graphene is a 2D sp^2^ hybridization honeycomb lattice of carbon atoms material, and it has excellent conductivity and acceptable biocompatibility (Bramini et al., 2018). When co-cultured with neurons *in vitro*, graphene could induce upregulation of cholesterol of neural membranes through a glia-like manner, while maintaining the morphology of neurons without obvious cytotoxicity (Kitko et al., 2018). Graphene was also able to regulate the extracellular concentration of potassium ions to enhance the electrophysiological function of neurons (Pampaloni et al., 2018). Cortical neurons cultured on poly (3-hydroxybutyrate) 3D scaffolds mixed with graphene nanoplatelets had stronger action potential signals, which were equivalent to the physiological state, and this construct reduced potentially toxic residues produced by traditional CVD in graphene as well (Moschetta et al., 2021). PCL scaffolds mixed with reduced graphene oxide nanoplatelets could maintain the solid phase of graphene for a considerable time and had similar degradation efficiency to common hydrogel scaffolds, and thus could be engineered in neuron regeneration (Sánchez-González et al., 2018).

Furthermore, the most distinct feature of neurons in forming neural networks is their orientation, the direction signals travel from the cell body to the axon and through synapses to the next neuron. The regulation of the direction of axon *in vitro* is a very intriguing research field, and this has particular bionics significance for spinal cord, which is a highly directional tissue. Primary neurons cultured on a microgrooved PLA nanosheet could generate thick neurite bundles along the direction of grooves, which was lot more well-constructed than dendrite growth on a flat nanosheet (Otomo et al., 2020). Such microgrooved structure also induced upregulated expression of postsynaptic membrane-related proteins and promoted neuronal differentiation and polarization. Uniaxial temperature gradient field crystallization of silk fibroin solution could produce silk scaffold with oriented channels, allowing neurons to align in oriented pores (Zhang et al., 2012). Regenerated silk fibers produced by straining flow spinning, with diameter of only 2 μm, facilitated directional migration and axon elongation of neurons (Mercado et al., 2020). However, the electrospun fibers either lack the interconnected pore, making the cells difficult to infiltrate and migrate, or present insufficient supporting strength. Therefore, the combination of highly aligned PCL electrospinning with 3D-printed PEG scaffolds could promote the adhesion and survival of neurons while inducing neuronal orientation (Lee et al., 2017).

### Advantages and limitations

Scaffolds exploit diverse biomaterials to directly provide a 3D growth environment for cells cultured *in vitro*. Exploration of novel biomaterials and revision of disfavored properties provide scaffolds with strong plasticity in biological and physical traits, manufacturing process, macro- and microstructure, and biochemical function, which also makes them valuable in a variety of aspects. In terms of the construction of the pathophysiological diseases model, scaffolds can induce the differentiation and maturation of neurons better than traditional culture systems and functionally restore the characteristics of neurons *in vivo*, beneficial to the research of neurodegenerative diseases and the development of therapeutic drugs. However, there is no universally recognized and an effective evaluation method for the 3D culture system at present. Most traditional assessment tools were developed for 2D cultures, which are not applicable in scaffold-based culture. As the promotion of 3D culture, future innovation of practical instruments designed for the 3D system could be expected. For treatment of diseases, scaffolds can carry cytokines and drugs that regulate the growth and function of neurons, and they can be used for tissue regeneration with tissue-like microstructure, developed as implants for the repair of CNS and PNS injuries.

## 3D bioprinting model

### Modalities

**FIGURE 3:**
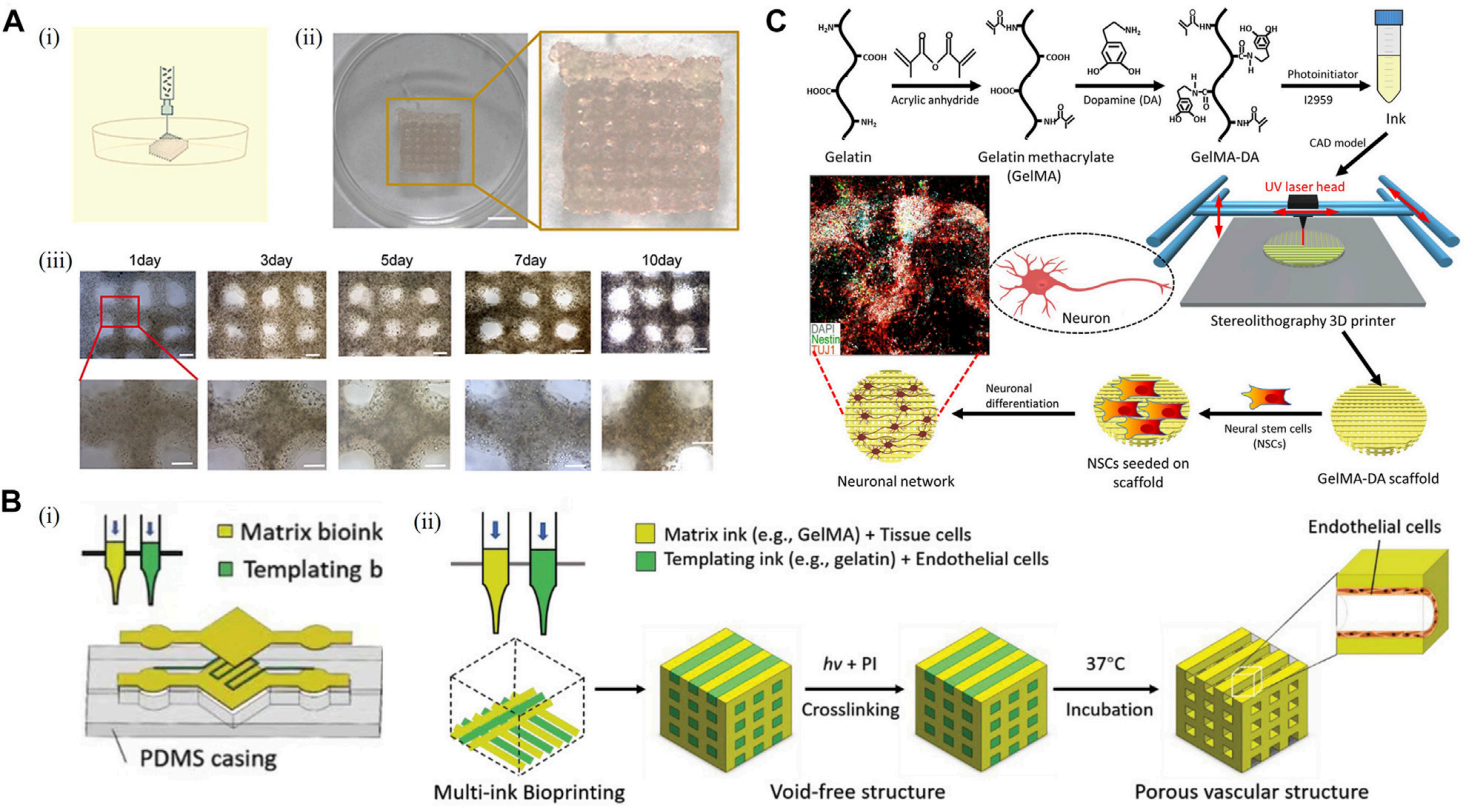
3D bioprinting model. **(A)** Establishment of a 3D bioprinted model. **[(A), i]** Schematic representation of the 3D bioprinting procedure. **[(A), ii]** Image of a 3D bioprinted model. Scale bar: 50 mm. **[(A), iii]** View of the *in vitro* model on days 1, 3, 5, 7, and 10 after 3D bioprinting. Scale bar: 1 mm. (Sun et al., 2020) **(B)** 3D bioprinted model for endothelialization and microfluidic perfusion using bioinks and sacrificial materials. **[(B), i]** Schematic illustration of a hydrogel-based microfluidic system generated with a sacrificial bioink used to template predefined channels. **[(B), ii]** Schematic illustration of the modified 3D bioprinting process, where a templating bioink loaded with endothelial cells for endothelialization and a matrix bioink are bioprinted side by side, followed by crosslinking of the matrix bioink and 37°C incubation to release the sacrificial bioink (Ouyang et al., 2020). **(C)** Schematic illustration of a 3D bioprinted model using dopamine-modified bioink. Adapted with permission. (Zhou et al., 2018) Copyright © 2018, American Chemical Society.

3D bioprinting is a novel additive manufacturing derived technology to construct highly ordered structures using cell-loaded bioinks. 3D bioprinting technology has gained rapid development in recent years due to its flexible choice of loaded cells and design freedom. Application of 3D bioprinting enabled the construction of centimeter-scale models with a predesigned structure. Therefore, the 3D bioprinting model is expected to restore the function of CNS *in vitro* and is significantly different from the traditional scaffold-based model. However, there are limited reports using 3D bioprinting technology to construct *in vitro* models containing primary neurons. Currently, the modality applied in 3D bioprinted primary neuron models is mainly extrusion-based bioprinting. Syringe-based mechanical structures are commonly used in extrusion-based bioprinting to continuously extrude cell-loaded bioink, constructing a predesigned macrostructure in a layer-by-layer fashion. Extrusion-based bioprinting has the advantages of convenient construction and flexible selection of bioink and cell types. However, its spatial resolution is limited so it is difficult to achieve the construction of fine microstructures (Cadena et al., 2021). In recent years, extrusion-based bioprinting technology has gained remarkable achievements in the fields of *in vitro* modeling and regenerative medicine. Using patient-derived tumor cells, 3D-bioprinted models were constructed for the screening of chemotherapy drugs, which paved the way for the realization of personalized medicine (Mao S. et al., 2020; Sun et al., 2020) (Figure 3A). Furthermore, 3D-bioprinted drafts loaded with functional cell line could partially replace organ functions and prolonged the survival of mice, showing promising potential for application in the field of regenerative medicine (Yang et al., 2021). Compared to traditional monocultures, cells embedded in 3D-bioprinted structures display distinct gene expression profiles, phenotypes, and drug responsiveness (Zhang et al., 2021). Compared to cell-free scaffold, biomaterials for bioprinting have more restriction on mechanics and fabrication. In terms of bioprinted neural cell models, primary neurons are so fragile and sensitive to shear force that they are vulnerable to damage in the 3D bioprinting process. Therefore, the selection of bioinks in constructing bioprinted primary neuron models is particularly important. As the biomaterials in which primary neurons grow, bioink determines the survival and function of neurons within the bioprinted models. In addition to the major characteristics suitable for cell culture as mentioned in the scaffold-based model section, bioinks used in 3D bioprinting are also required to have good printability and shape fidelity to adapt to the biofabrication process (Schwab et al., 2020). To be specific, ideal bioinks should possess satisfactory biocompatibility as well as appropriate mechanical properties and provide biochemical and structural support for the survival and growth of primary neurons. Meanwhile, optimizing rheological factors including shear-thinning behavior, viscoelasticity, and cells embedded in the bioink could improve the printability of bioinks. As a result, good biocompatibility and printability reduce the mechanical damage to primary neurons during the printing process and improve the viability of primary neurons. Appropriate mechanical properties such as stiffness and pore size that closely mimic the native microenvironment promote the growth of neurites. In addition to the selection of biomaterials, designing proper structures is also vital for restoring the internal microenvironment using 3D bioprinting technology. For example, ECM-based cell-loaded bioink and gas-permeable silicone ink were used to construct a multicellular system, in which glioblastoma cells were surrounded by endothelial cells within a microfluidic device. The model successfully restored the tumor microenvironment and functional BBB structures *in vitro* (Yi et al., 2019). Bioprinting with bioinks and sacrificial materials such as Pluronic F-127 was utilized to construct vascularized 3D structures (Ouyang et al., 2020) (Figure 3B), which was expected to establish perfusable models *in vitro* and contribute to the survival of 3D bioprinted transplantation grafts. Different 3D bioprinting modalities, the selection of appropriate bioinks and reasonable design of model structures make 3D bioprinted primary neuron models suitable for different application scenarios to bridge the gap with *in vivo* animal experiments and constructing transplantable tissues for traumatic injury repair.

### Bioprinted primary neuron model

Only a few articles have reported 3D bioprinting of brain-like structures so far. Using a gellan gum-based hydrogel, bioprinted primary neuron models were first constructed by hand-held bioprinting as a proof-of-concept (Lozano et al., 2015). As a bioink, gellan gum has an appropriate pore size, which can support the permeation of oxygen and nutrients, partially simulate mechanical properties in the body, and promote the survival of primary neurons and the extension of neurites. Although hand-held bioprinting, as the crudest printing method, relies on imprecise manual control of the printing structure, the successful construction of layered brain-like structure has proved that by adjusting the appropriate composition and concentration of bioink, primary neuron can withstand the shear force in the process of extrusion printing. With the progress of 3D-bioprinted primary neuron culture, a more perfect model has been successfully established. The neural-layered structures were successfully constructed by using alginate, gelatin, fibrinogen, and other biomaterials (Song et al., 2020). In order to improve the survival of primary neurons, the researchers also optimized nozzle inner diameter, printing speed, and other key printing parameters, providing valuable experiences for subsequent research. During *in vitro* culture, primary neurons in 3D neural-layered structures exhibited robust neurite sprouting and enhanced cell viability over 14 days. In addition, the excitatory postsynaptic potentials of 3D neural-layered structures in the primary neuron were successfully observed by multielectrode arrays, which could be significantly inhibited by neurotoxicity drugs such as tetrodotoxin. The aforementioned work has proven that 3D bioprinted primary neuron models can dutifully react to neurotoxic drug and have a potential application value for drug screening and pharmaceutical study. It is worth mentioning that biomaterials used in 3D bioprinting technology can significantly affect the characteristics and function of primary neurons in the model. For instance, modification of gelatin methacrylate with dopamine upregulates the expression of neural markers such as TUJ1 and MAP2 of primary neurons and enhances the differentiation of neural stem cells (Zhou et al., 2018) (Figure 3C). The application of functional biomaterials in 3D bioprinting technology can also be extended to the field of regenerative medicine. 3D bioprinting structures constructed by chitosan, hyaluronic acid, Matrigel, and other biomaterials have been proven to play a repairing role in rat spinal cord injury models (Liu et al., 2021). Furthermore, neurotrophin-3 and other compounds that promote nerve regeneration were incorporated in PLGA microcapsules, which could induce spatiotemporal manipulation of those compounds to construct spatially heterogeneous spinal cord injury repair graft with a higher degree of design freedom. The graft could directionally guide the growth of nerve cells and improve the motor function of spinal cord injury rats (Chiang et al., 2021).

### Advantages and limitations

The bioprinted primary neuron model is expected to construct a layered structure similar to the brain *in vivo*, building a multicellular system by loading different types of cells, and exploring the intercellular interaction mechanism in a 3D culture system. In addition, with the development of new technologies such as suspension printing and coaxial printing, the biofabrication window and printing accuracy of 3D bioprinting have been continuously expanded (Ma et al., 2020). In the future, the combination of 3D bioprinting and other biofabrication technologies may build brain-on-chip systems with fine structure and function *in vitro*. In addition, 3D-bioprinted primary neuronal systems have broad application prospects in drug development due to their high throughput potential and drug sensitivity. Using 3D bioprinting technology to restore the special structure of the CNS provides an important research tool for drug delivery across the BBB and other research fields. However, bioprinted primary neuron models still have drawbacks that imminently need to be improved, such as the optimization of biomaterials used in biological 3D printing. Different types of cells prefer specific growth environments provided by biomaterials. Currently, some compartments of the commonly used 3D bioprinting inks, such as calcium ions in sodium alginate-based system, or 3D bioprinting process, could lead to certain damage to primary neurons. Thus, it is an important subject for the development of bioprinted primary neuron models to determine the most suitable bioink for each type of neurons and the optimal constitution of bioink in the multicellular biological 3D printing system on the premise of satisfying printability (Fantini et al., 2019). For example, decellularized ECM and gelatin methacrylate as the emerging biomaterials for *in vitro* modeling and 3D bioprinting (Bae et al., 2021) are expected to be applied in the bioprinted primary neuron model as next-generation bioinks in the future. The construction of disease models using bioprinted primary neuron models, such as neurodegenerative diseases like Alzheimer’s disease and Parkinson’s disease, or co-printing with glioma cells to build *in vitro* models of CNS, will contribute to drug development and illustration of neuron-glioma interaction in the future. Due to the complexity of bioprinted primary neuron models, many observation and characterization methods, such as patch clamp technology, are not fully applicable to the bioprinted primary neuron models, which limit the application of 3D bioprinting models. The modification of traditional methodologies to make it compatible with 3D bioprinting is a key step to expanding the application scenarios of bioprinted primary neuron models. In addition, it is difficult to construct 3D bioprinting models of human primary neurons due to the numerous number of cells required to construct 3D bioprinting models and the limited source of human primary neurons. Finally, there is still a lack of comparison between bioprinted primary neuron models and other 3D culture systems, such as the microfluidic system and organoid system, and the differences in the growth status of primary neurons in different 3D culture systems have not yet been systematically evaluated. In a word, 3D bioprinting is a rapidly developing and emerging technology with broad prospects in neurobiological research.

## Microfluidic chip

### Modalities

Microfluidic chip is a complex culture of primary neuron, using microfluidic technology and microchips. In the chip, cells and tissues are grown in microchambers, and the overall microenvironment can be highly controlled. The simplest microfluidic chip is a single, irrigable chamber, in which one cell or a mixture of cells can be grown. In more complex designs, two or more chambers in the same chip are separated by membranes, channels, or gels to culture different cell types, with cells in each chamber either in direct contact or through secretions to transmit the intercellular interaction (Bhatia and Ingber, 2014; Phan et al., 2017). With advances in precision science and techniques, scientists have expanded from simple culture to regulation of cells by external controls to motivate their interactions and culture the cells in a more precise manner. The main application of this “precise culture” is the microfluidic chip. It can regulate a discrete neurite around a single neuron and the flow-controlled microdomain of some neuron microcircuits (Wang and Levchenko, 2009).

**FIGURE 4:**
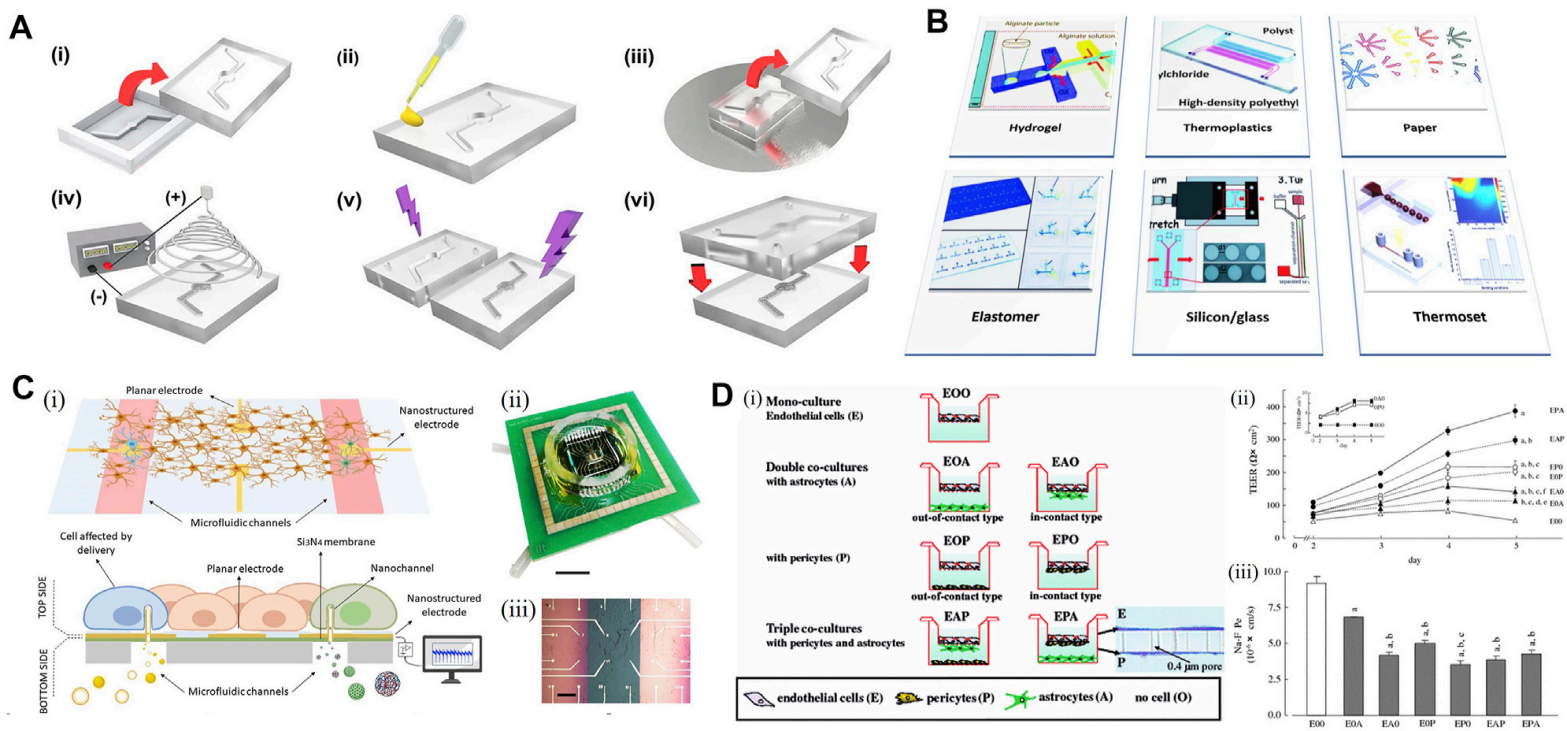
Microfludic chip for the *in vitro* model. **(A)** Schematic representation of the fabrication process of a PDMS-based chip. **[(A), i]** Top and bottom PDMS microfluidic channel substrates are formed by pouring PDMS mixtures into a mold and demolding it from the master mold. **[(A), ii]** Coating PDMS substrate with silver nanowires (AgNW) solution on top of the bonding interface. **[(A), iii]** Stamping PDMS substrate on spin-coated uncured PDMS layer forming an AgNWs-embedded uncured PDMS adhesive layer. **[(A), iv]** Direct electrospinning nanofibers to the bottom PDMS microfluidic channel substrate. **[(A), v]** Forming a free-standing polycaprolactone (PCL) nanofiber membrane on the channel area. **[(A), vi]** Integration of nanofiber membrane-deposited PDMS substrate with top PDMS microfluidic channel substrate by the free-standing PCL nanofiber membrane. Adapted with permission. (Rhyou et al., 2021) Copyright ^©^ 2021, American Chemical Society. **(B)** Schematic illustration of the materials used for the fabrication of microfluidic chips in neurodegenerative studies such as Alzheimer’s disease (Prasanna et al., 2021). Hydrogels serve as matrices for cell culture in microfluidic chips. **(C)** Microfluidic device with planar and nanostructured electrodes. **[(C), i]** Scheme of the device. **[(C), ii]** Picture of the device from the top side. **[(C), iii]** Top surface in detail, with feed lines and electrodes on thin silicon nitride membranes (pink) and bulk surface (green) (Bruno et al., 2020). **(D)** *In vitro* BBB model co-culturing rat brain capillary endothelial cells with pericytes and astrocytes. **[(D), i]** Schematic of BBB models. **[(D), ii]** Effectiveness of co-culture on the induction of transendothelial electrical resistance (TEER) in brain capillary endothelial cell monolayers of *in vitro* BBB models, with the left upper panel indicating a very low TEER of astrocytes (filled square, 0A0) and pericytes (open square, 0P0) cultured on the inserts. **[(D), iii]** Transendothelial permeability changes for the paracellular permeability marker in brain capillary endothelial cell monolayers of *in vitro* BBB models (Nakagawa et al., 2007).

Novel biomaterials have been significant for the development of microfluidic chip technology. PDMS is the most widely used material in microfluidic chip manufacturing. After the pattern is transferred to the photoresist through the mask, PDMS and curing agent will be mixed in a certain ratio, then pumped and poured onto the photoresist mold for solidification. The chip will be treated in the vacuum discharge plasma chamber with a glass slide followed by incision and punching, with a firm and irreversible bonding effect. The concave structure of PDMS forms a channel, and the bottom of the channel is sealed by the glass slide so that the chip could be transparent and easy to observe. Finally, PDMS will regain hydrophobicity after drying in a 105°F oven, which ensures the stability of the whole system (Figure 4A). Other biomaterials, such as polysilazane and gelatin, have outstanding characteristics in common (Asthana et al., 2006) that the materials are inexpensive and easy to mold and copy. Meanwhile, the specific biomechanical properties of PDMS have made it possible to adjust the neurons in the chip, related areas around neurons, and the neural network with a high degree of control precision (Taylor et al., 2010). This control, existing at spatiotemporal latitudes can be applied to neurons cultured in nanoliter with 3D terrain for precise operation. The high freedom of design can lead to customizing culture methods to solve new problems (Figure 4B). For instance, researchers have reported a selective approach to hippocampal neuronal guidance using 3D vertical nanorod arrays functionalized by a specific adhesion promoting molecule, polDL-ornithine (PDLO). They found that 90% of neural cells were orientated only on the combined PDLO/nanoparticle substrate (Amin et al., 2018). This successful induction of synaptic stability and cell activity on multilevel factors suggested the potential for designing nanostructured chips to control neuron growth in tissue engineering, neuronal repair, and drug development.

### Primary neurons on-chip

The specific application of the microfluidic chip mainly reflected in the following aspects. First, the controlled substrates have been used in a wide range of biological applications to regulate surface contacts and signals (Millet et al., 2010). In non-microfluidic models, these substrates could provide different preparation advantages, and for microfluidics techniques, an important application is that it can be used to model substrates to guide neuronal populations (von Philipsborn et al., 2007). The study was conducted to build a microchamber that could allow axons to grow through the tunnels connecting the chambers bidirectionally but with a designed, unidirectional bias. The result showed that the axons of the rat cortical neuron grew through the tunnels and connected to neurons in adjoining wells when being cultured in the chambers, with seventy-nine percent of burst transfers in the forward direction (Gladkov et al., 2017). This indicated that a simple, substrate-controlled neuronal circuit might be applied to develop *in vitro* models, for studying the function of cortical microcircuits and deep neural networks.

Second, the microfluidic chip can be used for some special cell co-culture models. In addition to the traditional implementation of differential shunt-controlled exposure, sample handling and collection, and cell-to-cell contacts, the cell source of these models could be neurons from different brain regions with their own specific microenvironments and surrounding cytokines, and they can be rigorously isolated in microfluidic systems to construct special models. At the same time, microfluidic chips make it easy to cross innervate slices of cells isolated from different brain regions through microchannels manufactured in the device. Finally, the microfluidic chip is a genuine 3D culture system *in vitro*. Different from the 2D culture that the surface of the substrate, providing the base for scattered neuron culture, it can define the terrain, which can more truly simulate the brain structure and thus, operates closer to the brain microenvironment dynamics. In a more mimic culture system, interactions between neurons or neurons and surrounding cells would also be more precise and biologically relevant.

There have been various kinds of research on the newly approached mechanisms of CNS activity, with the help of microfluidic chips. With the microfluidic system, scientists could build models with different chambers, and each one provides its function (Figure 4C). For those focusing on the mechanism of the neural network, the microfluidic chip helps to control the network more concisely, and make it easier to be regulated. To study the movement and transport of endosomal sorting complex required for transport (ESCRT) in axons of primary hippocampal neurons, scientists prepared microfluidic chambers to conduct the seeding, fluidic isolation, and lentiviral transduction of hippocampal neurons inside. On the aids of the live imaging of microfluidic separation axons, researchers were able to optimize the study of ESCRT protein dynamics (Birdsall et al., 2019). Similarly, a network patterned microfluidic chip was designed to evaluate the feasibility of constructing a 3D hippocampal neural network *in vitro*. The primary hippocampal neurons of neonatal Sprague–Dawley rats were isolated and cultured, and then inoculated in a microfluidic chip for culture (Kong et al., 2019). As a result, the chip was successfully constructed in single and multi-channels, with spontaneous firing signals of hippocampal neuronal networks detected at 7 days of culture. The result indicated that neuronal networks already possessed biological function, which provided solid evidence that hippocampal neuron network could grow within limited pathways; therefore, making it possible to construct a 3D neuron network *in vitro*.

The co-culture models with microfluidic chips have various subtypes. Take the neurovascular unit (NVU) as an example, which is a new concept born in 3D model based on the traditional model consisting of brain microvascular endothelial cells and neurons (Wang et al., 2016). The new model added astrocytes to the co-culture system and was designed to use neurotoxic drugs to test cell viability. By calculating the half-maximal inhibitory concentration (IC50) and intracellular calcium concentration under the exposure of Pb^2+^ and Al^3+^, both the two models showed distinct permeability compared to the control, which suggested that they might be better models in selecting neurotoxicity drugs and representing the BBB.

A particularly important model applying microfluidic chips is the construction of BBB *in vitro*. BBB is one of the most important structure surrounding human brains, which could be a potential good application area for NVU model. BBB has become a rising research topic because of its low permeability leading to lots of failure for CNS drug delivery. Thus, scientists have been working on a model highly mimicking the cellular structure and microenvironment of the BBB. The setups were first using the classical Transwell assays, but the models did not show enough efficiency with monocultures or co-cultured systems (Cecchelli et al., 2014; Wolff et al., 2015). The reason could be that the two phases separated by the BBB have relatively different characters, from cell components to the cytokine and the microenvironment around neurons and pericytes. With the novel approaches using microfluidics, it has been the first time for the researchers to divide a culture model into two isolated flow systems, while they could still be regulated to interact with each other, which is very close to the real condition in human brains. Rat brain vascular endothelial cells (RBECs) were used to co-culture with astrocytes and pericytes to study the effect of cell composition on the resulting barrier properties. Researchers found that the transendothelial electrical resistance (TEER) increased almost two-fold when the pericytes were in contact with the endothelial cells instead of the astrocytes (Nakagawa et al., 2007) (Figure 4D), which has proven the previously mentioned statement. Therefore, despite the model has not yet reaching beyond the proof-of-concept stage, it has intended to become an anticipated development of BBB research with great potential.

### Advantages and limitations

The microfluidic chip has combined the techniques previously developed by scientists to study the complicated cell system, such as liquid exchange with special flasks, density control, and optical imaging with the usage of hanging drop and chamber. With new biomaterials they could be put together on a small mold, to assemble all the abilities and to make neurons easier to cultivate. This has provided the model with superior features, such as lower water permeability, thermal stability, scalability, and biocompatibility (Millet and Gillette, 2012). Another important factor that cannot be avoided is its low cost and convenience. After generating a standard mold, the microfluidic device can be easily replicated with inexpensive reagents on the hood or bench, greatly reducing experimental costs and increasing the efficiency of neuron culture. However, the design of the first standard mold is relatively difficult and is in need of highly skilled researchers. Producing microfluidic chips also has high requirements for equipment, which is not easyly qualified by most laboratories. This situation is certainly being improved in the coming years, so there may be increasing applications of the microchip in future.

## Discussions

The brain is an organ with complex microstructure and refined function regulation. Neurons are the most basic structural and functional units of the CNS, which are challenging to be cultured *in vitro* and easily affected by changes in the external environment. Primary neurons are directly obtained from animals or humans and can represent the features *in vivo*, which has a great application value in disease modeling, research of mechanisms, and regenerative medicine. Therefore, more attention has been paid to constructing *in vitro* models using primary neurons. Since the traditional 2D culture of primary neurons cannot fully meet the current research needs, novel *in vitro* models suitable for specific research scenarios are constantly developed to restore the *in vivo* environment more accurately. Particularly, biomaterials play a vital role in the construction of advanced primary neuron models. Different models, including the *in vivo* and *in vitro* models, can help in exploring the mechanism of disease occurrence and progression, as well as potential drug screening and therapy development.

In this review, the 2D *in vitro* model, *ex vivo* culture system, spheroid, scaffold-based culture system, bioprinted primary neuron model, and microfluidic chip are introduced. Different models are heterogeneous in terms of biomaterials used, modeling methodology, characteristics, and advantages, so it is critical to select appropriate models for the experimental design and functional realization **(** Table 1 **)**. 2D *in vitro* models include traditional 2D culture, co-culture system, Transwell culture, and other methods to construct a multicellular system, which can realize direct or indirect contact between primary neurons and glial cells, immune cells, and other cells. The 2D *in vitro* model is convenient to construct and flexible to design. The novel 2D *in vitro* model has been widely used in the study of cell–cell interaction and other mechanisms. However, because the 2D model cannot simulate the 3D structure in the CNS, it is not able to fully restore the *in vivo* environment as good as other 3D models. *Ex vivo* culture is a simple and effective model of primary neuron, which was adopted to reproduce the distribution of cells *in vivo*. At present, the commonly used *ex vivo* culture methods include slice culture, such as cerebral cortex slice and hippocampus slice, and tissue culture *in vitro*, mostly primary hippocampus culture. The advantage of this model is that it can maintain cell diversity, 3D neuronal networks, and intact cell connections while requiring no additional technology and new biomaterials compared to other new models. Spheroid-based primary neuron models are developed by embedding neurons in functional biomaterials such as Matrigel or using low-attachment surfaces. Compared with the traditional culture model, the spheroid-based primary neuron models significantly improved the viability of primary neurons, with significant differences in the cell morphologies and phenotypes of the primary neurons. As the simplest 3D culture model, primary neurospheres can partially reconstruct 3D structures *in vivo* and have the potential for high throughput applications. Scaffold is the most direct way to establish the 3D primary neuron culture system. Biomaterials and various physical or chemical methodologies are used to construct reasonable structure in order to facilitate neuron attachment and growth. In general, the scaffold-based culture can maintain the morphology and function of neurons *in vitro* and has higher plasticity, according to different application scenarios. Scaffolds can induce the differentiation and maturation of neurons *in vitro*, direct the orientation of axons, and promote the formation of functional neuronal networks. In addition, scaffolds may be used as implants to regulate the growth and development of neurons *in vivo*, and possess the potential for treatment and repair of neurological diseases. Bioprinted primary neuron models are constructed by extrusion-based bioprinting, droplet-based bioprinting, laser-assisted bioprinting technologies, and bioink loaded with primary neurons. Due to its high freedom of design, the bioprinted primary neuron models can simulate complex structures of the CNS such as layered structures and the BBB *in vitro*. This model has the potential for high throughput applications and can be used to construct uniform 3D models with high efficiency and grafts for the repair of central and peripheral nervous system injuries, showing good therapeutic effects in animal models. The microfluidic chip is a complex culture of primary neurons with the microfluidic system. The novel device has allowed delicate regulation of the extracellular environment, synapses, and neuronal networks. The biomaterial commonly used in microfluidic chips is PDMS, as well as other hydrogels and polymers, all of which are low cost and reproducible, giving the microfluidic chip a relatively high-cost performance. Other advantages include a wide range of applications, high throughput potential, excellent versatility, thermal stability, biocompatibility, high design freedom, and fine adjustment. Microfluidic chips also have the prospect to develop complex neurovascular unit with a functional BBB and physiological fluid dynamics.

**TABLE 1:** Overview of *in vitro* model of primary neurons, with their features, advantages, and limitations.

*In vitro* model	Feature	Representative biomaterial	Advantage	Limitation
Novel 2D *in vitro* model	Multicellular system cultured on 2D surface, with direct or indirect contact	Poly-lysine coated plate	Easy to establish	Cannot fully restore 3D morphologies and functional characteristics *in vivo*
			Design flexibility	Lacks spatiotemporal arrangement
			Well-recognized characterization methods	
*Ex vivo*	Brain slice cultured on thin film inserts at gas–liquid interface	Porous membrane	Maintain cell diversity, 3D neuronal networks, and intact cellular connections	Limited culture duration
			No technological or material requirement	Difficult to standardize
Spheroid	Cell clusters formed by aggregation and division	Matrigel or low-attachment plate	Partially reconstruct 3D structures	No control on composition or arrangement of cells
			High throughput potential	Batch-to-batch heterogeneity
			easy to construct	
Scaffold	Cells seeded and cultured on hydrogel or fibrous structures	Natural and synthetic polymers	Plasticity of ECM characteristics	Biocompatibility
			Simulation of *in vivo* microstructure	Technological difficulty in production
			Sustained release of cytokines or drugs	
			Induction of neuronal network formation	
3D bioprinting	Deposition of cells embedded in bioink as designed structure	Hydrogels with controlled gelling and printability	Precise control over spatial arrangement of cells	Shear stress damage to neurons
			High throughput potential	High cost
Microfluidic chip	Neurons cultured in microfluidic device with multi-chamber design	PDMS	Fine regulation of environmental factors	High requirements for equipment
			High reproducibility	Difficulty to develop physiologically relevant design
			High throughput potential	

Over recent years, *in vitro* neuron models have witnessed the advancement from the traditional 2D culture system to the novel 3D culture system. However, one important issue of 3D cultured is the means for analysis. Genetic modification is helpful in the study of biomechanism, yet limited conductivity and permeability of the 3D system to vectors hinder the application of this tool. Biochemical blotting and sequencing require extraction of nucleic acids and proteins from cells cultured in 3D models, which is often preceded by deconstruction of 3D structures to release cells. This process could potentially alter the features of cells, especially for cells as delicate as neurons. Meanwhile, it is difficult to use the traditional patch clamp technology for extensive detection of the 3D neural network, and multielectrode array also has its limitations of conductivity and instruments, which could be partially solved by a 3D multielectrode array (Lam et al., 2021). Calcium fluorescence staining for electrical signals imaging also requires great systemic transparency and high spatiotemporal resolution. Using lattice light-sheet microscopy to conduct calcium imaging on 3D-cultured neurons can achieve high-speed volumetric imaging with less phototoxicity (Chen C. Y. et al., 2019). In terms of data analysis methods, local neural functional networks can be constructed, according to the intensity and consistency of calcium signals in neurons (Dingle et al., 2020).

### Biomaterial—neuron interaction

Biomaterials are unavoidable in the construction of *in vitro* models, regardless of the complexity of the chosen model, from the most common Petri dish to delicate microfluidic systems. Ideal biomaterials should provide a suitable physical environment and good biocompatibility to improve the survival of primary neurons. Appropriate selection of biomaterials, and sometimes, modification of surface chemistry to provide cellular binding sites is the first step for primary neuron culture. Stable form for long-term culture and suitable mechanics for biofabrication are also necessary, which requires both plasticity and constancy. In terms of structure, micropatterning, oriented microgrooved structures, or microfilament scaffolds were used to construct *in vitro* models conforming to histological arrangement, while Transwell and microfluidic are used to construct multi-chamber structures such as the BBB and NVU. In terms of function, the adhesion of neurons was enhanced by coating of poly-lysine, modification of surface chemical group, and adjustment of microstructure. Changing the physical and chemical properties of the material, such as stiffness and electrical conductivity, could make it more suitable for the growth of neurons. In the future, biological properties of materials may be further enhanced by immobilizing bioactive substances into materials to regulate neurons, or by mixing stromal cells such as glial cells as a support system for neuron culture. Continuous optimization of the relationship between biomaterials and neuron cells through combination and modification of various biomaterials is vital, which demands the progressive understanding of the interaction mechanism between neuron and matrix or stromal cells. In conclusion, the selection of appropriate biomaterials and construction methods to restore the structure and function of neuron *in vivo* is the basis for establishing disease models and repairing nerve injuries.

### Applications in disease modeling and regenerative medicine

Disease modeling is one of the most important applications of primary neuronal culture. Many studies on the simulation of pathological states have been reported, such as ischemic stroke and traumatic injury. These models illustrate the processes of disease occurrence, and many treatments and drugs have been evaluated through them. Biofabrication technologies, such as 3D bioprinting technology and microfluidic chip, may be used to build complex structures such as NVU, providing better models for pathological research. Meanwhile, neurodegenerative disease is affected by many factors such as environment and genes, and its pathological process is mostly irreversible, which poses a serious threat to the health of human beings. There is still a lack of satisfactory models for neurodegenerative disease *in vitro* and *in vivo*. The main construction methods of current disease models are mainly based on environmental induction, which cannot fully represent the situation *in vivo*. With the aid of molecular biological manipulation technique, using primary neurons derived from disease model animals, or simulating pathophysiological states, can help the research on disease mechanisms and drug development. The combination of primary neural cells derived from patients and the 3D culture system is expected to construct a reasonable model of human neurodegenerative disease, providing a valuable tool for the study of pathogenesis and possible treatment. Questions remain regarding limitations on the source of human neurons as well as ethical concerns, so the combination of biofabrication methodologies and trans-differentiation technologies may be one of the potential solutions. The use of primary neural cells or neural stem cells for the repair of central and peripheral nervous system injuries is one of the current research hotspots. Neural cells can promote nerve repair by paracrine and other manners, biomaterials developed on basis of this mechanism can guide nerve regeneration and provide a suitable growth environment for neural cells. Transplants loaded with functional biomaterials can also promote neurogenesis and other repair processes (Chai et al., 2021), showing good therapeutic effects in a variety of animal models including brain injury and spinal cord injury. In regenerative medicine, the *in vitro* model of primary neurons as an important tool can provide supplementary validation and theoretical basis for animal experiments.

### Future prospectives

To our knowledge, this is the first review focused on models of primary neuron culture and how biomaterial contributes to each model. With the development of a variety of modeling techniques and the gradual deepening of the comprehension of the CNS, the primary culture of neurons has become increasingly complicated. The requirement for high representative will limit the potential of high throughput application. Therefore, suitable models should be selected for different purposes. For now, many current 3D culture systems lack well-recognized characterization methodologies, and the innovation of assessing methods in 3D culture systems can expand their application (Boutin and Hoffman-Kim, 2015). From the perspective of cell origin, primary neurons are closer to the state of neural cells *in vivo*. However, it is difficult to obtain human-derived cells, and perhaps iPSC-induced differentiation technology combined with patient-derived cells can be applied to accelerate the individualized study of diseases. In the future, with the improvement of biomaterials and technologies, *in vitro* neuron models can explore the mechanism of diseases, develop new drugs, repair tissue injury, and bring hope to patients with various CNS diseases.

## Conclusion

We have made an integral retrospect of the various models emerged in research with primary neurons, especially the novel one using innovative biomaterials in the past decade. These models could be divided into 2D culture model, *ex vivo* model, spheroid model, scaffold model, 3D bioprinting model and microfluidic chip, each of which has its own advantages and limitations, representing a specific stage of the development of primary neuron culture, 2D culture is the classical method of cultivation, but with new biomaterials coated Petri dishes, a precursor of 3D culture system is fledgling. The *ex vivo* model fills the vacancy of *in vitro* models with the help of PTFE-made porous membrane. With different biomaterials concerning biocompatibility, stiffness, conductivity, and microstructure, various 3D scaffolds have been fabricated for better function and morphology. Biomaterials with proper printability and shape fidelity enable 3D centimeter-scaled models with predesigned structures. PDMS, finally, has brought a revolution to the craftsmanship of microfluidic chips, making it an outstandingly precise model for multi-system culture, and with such retrospect, we can summarize the crucial point on how progression on biomaterials can lead to the development of primary neuron culture, and thus, provide a prospect on the future directions of this significant research field.
